# The Mississippi Valley Medical Association

**Published:** 1891-11

**Authors:** 


### THE MISSISSIPPI VALLEY MEDICAL ASSOCIATION.

The seventeenth annual meeting of this famous medical body was held in St. Louis, October 14, 15, and 16, 1891. Though this Association has been noted for large and enthusiastic meetings, the writer was told that the late one was the largest ever held, and that the enthusiasm was never greater. The registration list showed about three hundred names recorded, and this alone indicates the activity, life and prosperity of the Society. There are so many medical organizations in this country, that it is a wonder how they are all kept alive. It is a marvel to find men, whose time is money, and whose capital consists of brains and time, willing to travel across the continent, and from gulf to lakes, for the purpose of mingling with their professional brethren, and exchanging thoughts with each other on important medical questions. In the Southwest, particularly, there are several subordinate medical associations, made up of a union of two or more States, or of portions of States, in which latter case they are called District Medical Societies, so that the number is almost without limit ; nevertheless the enterprising physicians of the Mississippi Valley, appreciating the importance of frequently gathering together and touching elbows on questions that are agitating the medical world —questions of improved sanitation, of clean surgery and advanced midwifery, questions which relate to the prolongation of life, to the alleviation of human suffering, questions which make the race better, stronger, and purer—the physicians, we repeat, of this great and fertile region are not slow to grasp any and every opportunity to advance the science and art of medicine, to which they all seem to be so devotedly attached. The Association was particularly fortunate in the selection of its amiable and accomplished President, and Dr. C. H. Hughes, of St. Louis, will long be remembered for the strength and popularity of his administration of the affairs of this Association on this occasion. Another fortunate circumstance, that contributed not a little to the success of the meeting, was the selection of Dr. I. N. Love as Chairman of the Committee of Arrangements. It is easy to be seen that Dr. Love is the most popular physician of St. Louis, and this may be said without disparagement to any other. For a man of his years to have acquired his position, both professionally and socially, to be able to direct large affairs on such a large scale, and to actually rise head and shoulders above all others in such an important medical enterprise, is alone evidence of his magnetism, his amiability, his talent, and his goodness.

The receptions of Dr. Hughes, Dr. Bond, and Dr. Love were brilliant social events, marked for the beauty, fashion and wealth that gathered in their salons, and contributed not a little to strengthen the attachments which grow out of such a meeting. The banquet, on Thursday, at the Lindell Hotel, was one of the most brilliant medical feasts that has ever been given in this country. It was presided over by the talented and handsome Governor of Missouri, the Hon. David R. Francis, and Dr. I. N. Love acted as toastmaster, which is a sufficient guarantee of the brilliancy of the intellectual part of the feast. A number of toasts were responded to, and the guests lingered around the table until four o'clock A. M. Taken all in all, judged from any point of view, the seventeenth annual meeting of the Mississippi Valley Medical Association must be regarded as a pronounced success.

Dr. Chas. A. L. Reed, of Cincinnati, was elected President for the ensuing year, and the next meeting will be held in Cincinnati, in October, 1892.

---

STATE Board of Examiners—" Of what college are you a graduate? "

Candidate—" Of none. I thank God my intellect was never warped by college training."

Board—" Then you thank God for your ignorance ? "

Candidate—" You can put it that way if you choose."

Board—" Yes, and we find you have a great deal to be thankful for."—*Kansas Medical Journal.*

